# Papers Expected

**Published:** 1911-09

**Authors:** 


### Papers Expected

In the next issue, will be begun a series of articles giving in classified form, a list of new drugs accepted by the Council of Pharmacy of the A. M. A.

At about the time this notice appears, several of our old-country friends will be laughing over our attempt to write their languages. We expect, in reply, a number of short original papers which will be translated for exclusive publication in the BUFFALO MEDICAL JOURNAL, and of whose value there can be no doubt.

The rapid increase of state and municipal legislation requiring reports of contagious disease, and the like, places a considerable burden on the medical profession which they are willing to bear but which, through ignorance of the law, they sometimes fail to assume. We are promised a concise statement, by an authority, of the duty of physicians in this regard. This article will relieve the conscientious physician of worry and trouble in ascertaining what is required of him.

